# Obstructive jaundice due to a blood clot after ERCP: a case report and review of the literature

**DOI:** 10.1186/s12876-018-0898-4

**Published:** 2018-11-03

**Authors:** Yangbei Zhu, Shuling Wang, Shengbing Zhao, Lin Qi, Zhaoshen Li, Yu Bai

**Affiliations:** 1 Department of Gastroenterology, Changhai Hospital, Second Military Medical University, Shanghai, China; 2 Department of Gastroenterology, Shanghai Eighth People’s Hospital, Shanghai, China; 3 Company Six, Student Brigade, Second Military Medical University, Shanghai, China

**Keywords:** Endoscopic retrograde cholangiopancreatography (ERCP), Common bile duct (CBD), Endoscopic sphincterotomy (EST), Blood clot

## Abstract

**Background:**

Endoscopic retrograde cholangiopancreatography (ERCP) is one of the most frequently performed procedures for the treatment of biliary-pancreatic diseases. The most frequent complications of ERCP include pancreatitis, haemorrhage, perforation and cholangitis. While post-ERCP biliary bleeding leading to biliary obstruction is rare.

**Case presentation:**

We herein report a case of exceptional post-ERCP cholangitis due to a blood clot in the common bile duct (CBD). This case involves a 75-year-old woman with a history of recurring upper abdominal pain. Abdominal computerized tomography (CT) revealed dilatation of the extrahepatic bile duct with stones at the lower CBD. After ERCP, clearance of stones was obtained. The post-ERCP course was symptomatic with upper abdominal pain and a significant increase in cholestatic parameters. A second CT scan demonstrated a markedly dilated biliary tree with a longitudinal high-density image at the middle CBD. The patient was successfully treated with a repeated ERCP, and a blood clot was extracted. We also present a review of the literature published between 1985 and 2016 in PubMed. Four similar cases were reported during this period from France, Turkey, the USA and the UK, separately. Our case is the first reported in China.

**Conclusions:**

Post-ERCP biliary bleeding leading to biliary obstruction is rare. We describe a rare case of post-ERCP cholangitis due to a blood clot in the common bile duct (CBD), which is consistent with most clinical presentations of similar cases already described. An analysis of the possible pathophysiological mechanisms and a review of the current literature are provided. We attempt to attract clinicians’ attention to the differential diagnosis of post-ERCP obstruction. The complications might be severe or even fatal. The diagnosis of blood clot is based on clinical and laboratory data, particularly imaging. Repeated ERCP is often necessary and effective.

## Background

Currently, endoscopic retrograde cholangiopancreatography (ERCP) is minimally invasive and is one of the most frequently performed procedures in the treatment of biliary-pancreatic diseases. If the procedure is performed by experienced professionals, the total complication rate of ERCP is approximately 5% [1, 2]. The most frequent complications of ERCP include pancreatitis, haemorrhage, perforation and cholangitis. The rate of haemorrhage after ERCP has been reported to be as low as 1.3%, and the rate of post-ERCP cholangitis is 1% or less.

In this case report, we describe a rare case of post-ERCP cholangitis due to a blood clot in the common bile duct (CBD), which is consistent with most clinical presentations of previously reported similar cases. An analysis of the possible pathophysiological mechanism and a review of the current literature are presented.

## Case presentation

**Fig. 1:**
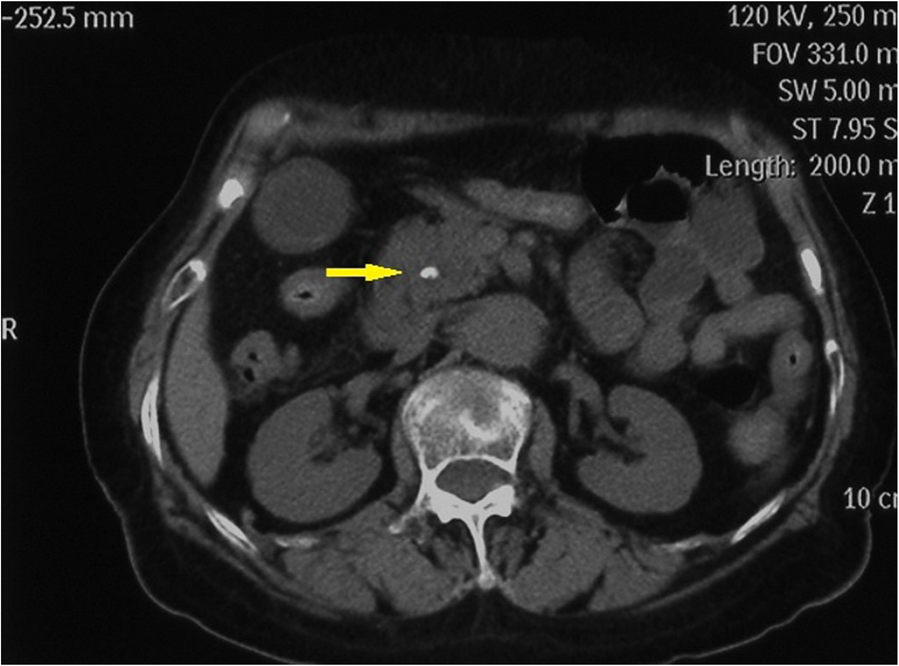
An abdominal computerized tomography (CT) scan demonstrating dilatation of the extrahepatic bile duct with a stone at the lower CBD

**Fig. 2:**
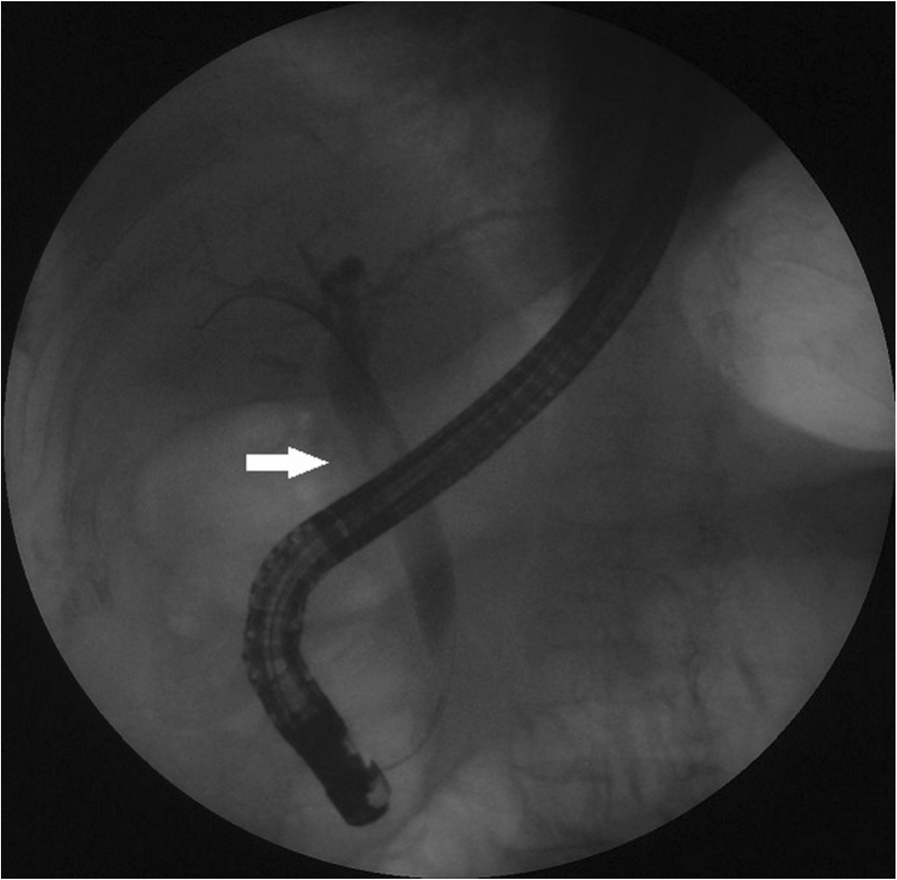
An ERCP revealed a dilated common bile duct with a round filling defect (arrow)

A 75-year-old woman was admitted to the hospital with abdominal pain, nausea and vomiting for 3 days. She did not drink alcohol, and there was no clinical or biochemical evidence of primary liver disease or coagulopathy. Physical examination revealed mild tenderness in the right upper abdominal quadrant. Laboratory tests revealed that the percentage of neutrophils (N%) was 80.3% (50–70%), alanine aminotransferase (ALT) was 192 U/L (< 64 U/L), aspartate aminotransferase (AST) was 66 U/L (< 64 U/L), γ-glutamyl transpeptidase (γ-GT) was 197 U/L (< 47 U/L), and all other laboratory parameters were normal (e.g., haemoglobin and platelet counts, prothrombin time, and renal function). An abdominal computerized tomography (CT) scan demonstrated dilatation of the extrahepatic bile duct with a stone at the lower CBD and sludge in the gallbladder. (Fig. 1) Bile duct cholangiopancreatography revealed a dilated CBD (10 mm in diameter) with a round filling defect (8 mm in diameter) (Fig. 2). Balloon dilation (10 mm in diameter) of terminal CBD after a 5-mm long sphincterotomy for extraction of the stone was uneventful. Unfortunately, she presented with cholangitis and a significant increase in the percentage of neutrophils (94%) and cholestatic parameters (total bilirubin 111.1 μmol/L (2–18 μmol/L), direct bilirubin 81.3 μmol/L (< 7 μmol/L), ALT 465 U/L, AST 538 U/L, and γ-GT 634 U/L) after 3 days.

**Fig. 3:**
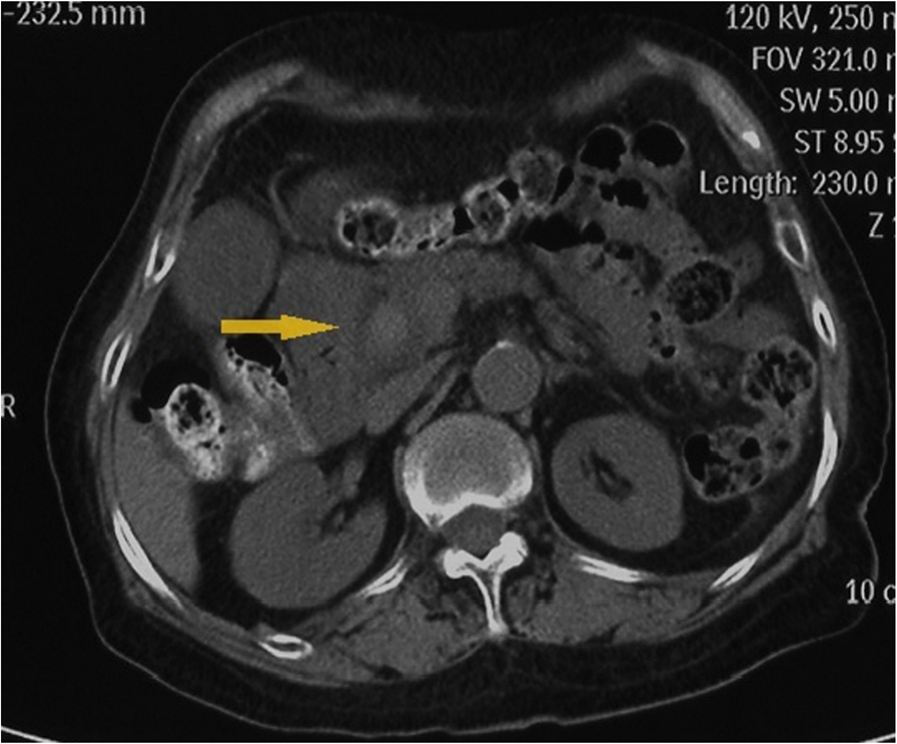
A high-density image of the middle CBD with a markedly dilated biliary tree on the second CT

**Fig. 4:**
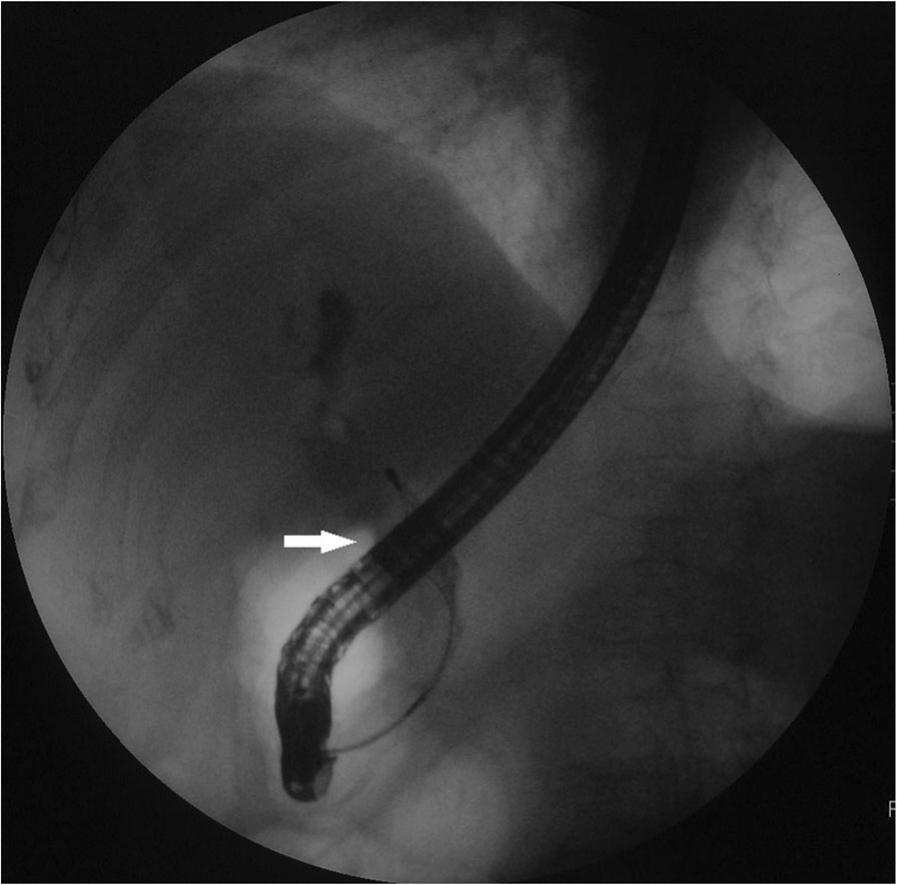
A repeated ERCP revealed a dilated common bile duct with a long filling defect. (arrow)

**Fig. 5:**
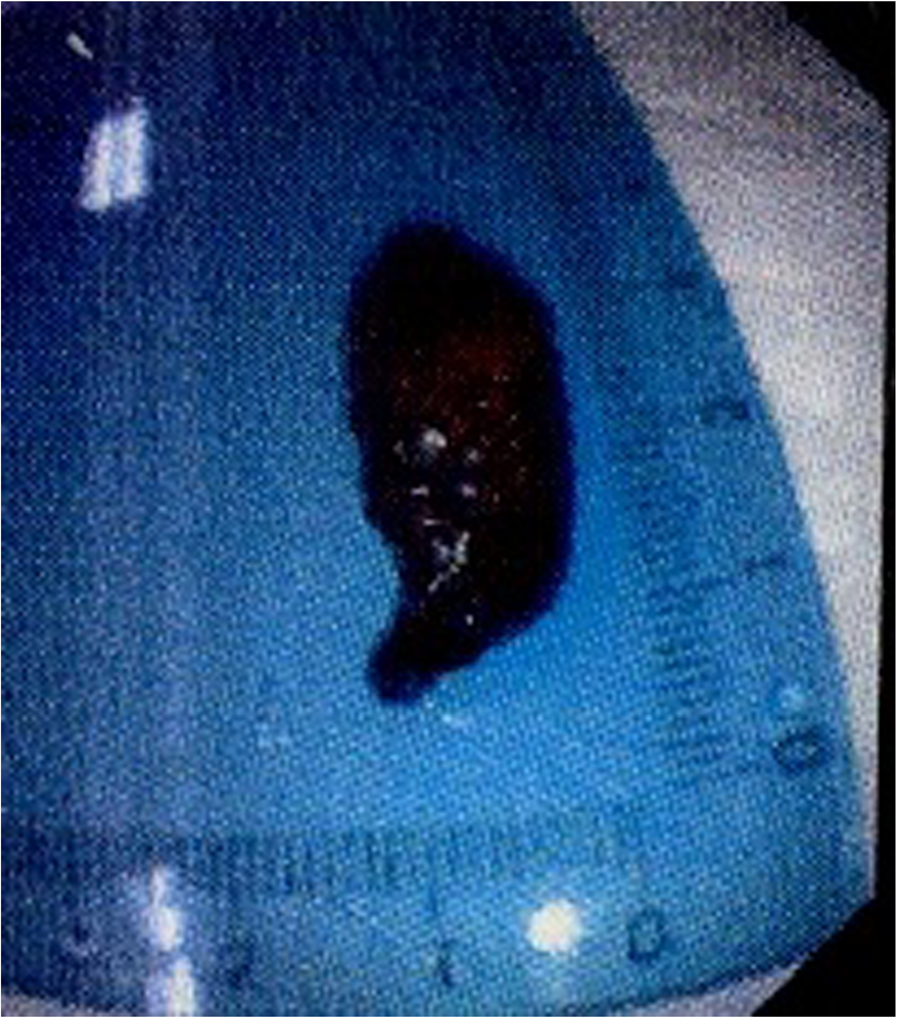
A blood clot (maximum diameter 3.5 cm × 1.0 cm)

**Fig. 6:**
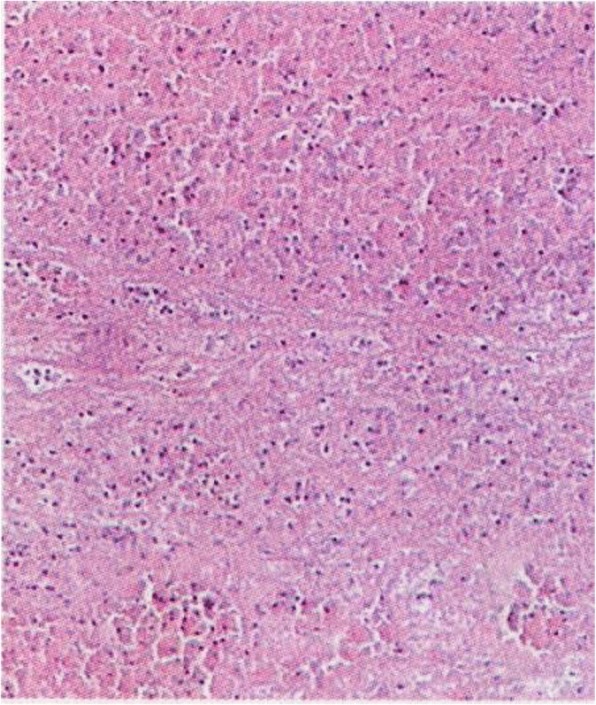
Histopathological examination revealed that the clot was composed of massive red blood cells and white blood cells with fibrin exudation and tissue necrosis

A high-density image of the middle CBD with a markedly dilated biliary tree was revealed on the second CT (Fig. 3). Thus, ERCP was repeated. A long filling defect was noted in the dilated common bile duct (Fig. 4), and a blood clot (maximum diameter 35 mm × 10 mm) was extracted with a basket (Fig. 5). Then, an endoscopic nasobiliary drainage (ENBD) tube was inserted into the CBD to ensure continued biliary drainage. Two days later, her temperature returned to normal, and abdominal pain was relieved. Histopathological examination revealed massive red blood cells with white blood cells and tissue necrosis (Fig. 6). After the treatment, she recovered and was discharged without any other complication.

## Discussion and conclusions

ERCP has been well established for the management of biliary and pancreatic disorders. Although ERCP is a relatively safe procedure, complications cannot be avoided in the performance of endoscopic procedures. It is important to recognize the adverse events and the risk factors to minimize the severity of complications. The potential complications of stone extraction involve pancreatitis, haemorrhage, infection and perforation, with a total incidence rate of approximately 5% [1, 2]. The rate of haemorrhage after ERCP has been reported to be as low as 1.3%, and the rate of post-ERCP cholangitis is 1% or less. As shown by multivariate analysis, definitive risk factors for post-ERCP bleeding includes coagulopathy, anti-coagulation within 72 h of sphincterotomy, cholangitis before ERCP, bleeding during initial sphincterotomy and lower ERCP volume. Other possible risk factors include cirrhosis, dilated common bile duct, papillary stenosis, precut sphincterotomy and common bile duct stone. Although But the reason for post-ERCP biliary bleeding leading to biliary obstruction is rarehasn’t be described, we seek to analysis the risk factor and attract clinicians’ attention to the differential diagnosis of post-ERCP obstruction.

After reviewing the literature in PubMed between 1985 and 2016, four cases of biliary blood clot caused by stone extraction have been reported [3–6] (Table 1). In 1997 [3], Mosenkis BN first reported post-ERCP cholangitis caused by a biliary blood clot. Due to slow emptying of contrast material, endoscopic sphincterotomy (EST) (2 mm) was performed during the initial procedure. However, their patient returned due to cholangitis after 3 days. A clot as large as the entire CBD was extracted after a further ERCP, and she recovered 1 week later. In 1999 [4], Aftab Ala observed a 4-mm stone at the ampulla with non-dilated CBD in the patient’s first cholangiography. To retrieve the stone, a 5-mm long EST was performed. The patient returned 4 days later with cholangitis and bleeding (haemoglobin 4.8 g/dL). ERCP was repeated 9 days after the initial ERCP, and a 3.5 cm blood clot was retrieved. The patient recovered after 3 weeks. In 2012 [5], Ergül B reported a 61-year-old woman’s ERCP completed with EST and biliary stent placement due to the difficulty in contrast agent drainage. She developed cholangitis on the next day. A markedly dilated biliary tree was noted on ultrasonographic examination, and an emergency ERCP was performed again. She recovered well after 3 days. In 2013 [6], the initial ERCP in Maroy B’s study reported a 12-mm diameter stone above the pancreas. The intrapancreatic bile duct was only 7 mm. To extract the stone, a balloon was dilated to 13 mm. The patient developed pancreatitis and angiocholitis with septicaemia in the following days. Immediate plastic stenting through a clot did not stop multisystemic failure and multiple liver abscesses. He finally recovered after 2 months of hospitalization.

**Table 1:** Published reports on endoscopic retrograde cholangiopancreatography in patients with blood clots

Ref.	Year	Patients Age/ Sex	Procedure	Anatomical abnormality	Recurrence time after the first procedure	Complication(s)	Treatment	Recover time	Outcome
Mosenkis BN Ref 3	1997	68/F	ERCP+EST	a mild dilation CBD	3 days	cholangitis	ERCP+ balloon dilation	1 week	alive
Aftab Ala Ref 4	1999	27/M	ERCP+EST	normal CBD	4 days	cholangitis and bleeding	ERCP+EST + stent placement	3 weeks	alive
Ergül B Ref 5	2012	61/F	ERCP+EST + stent placement	a blunt end of the CBD at the level of the ampulla Vater	1 day	cholangitis	ERCP+ balloon dilation	3 days	alive
Maroy B Ref 6	2013	45/M	ERCP+balloon dilation	an intrapancreatic bile duct	2 days	pancreatitis, angiocholitis, multisystemic failure and liver abscesses	ERCP+ stent placement	2 months	alive

*ERCP* endoscopic retrograde cholangiopancreatography

*EST* endodcopic sphincterotomy

All the five cases including ours had post-ERCP cholangitis due to a blood clot in the CBD. We found that the blood clot in the CBD was caused by delayed bleeding after ERCP with a time interval varying from 24 h to 4 days. Extraction of the stone from the common bile duct should explain the haemorrhage. Therefore, as illustrated in the summary of previously reported cases, ERCP with sphincterotomy is considered a definite risk factor for bleeding. Four of five cases reported EST in the initial procedure, but EST does not appear to be related to the length of the incision [7].

The mechanism by which haemobilia leads to a blood clot remains uncertain. After analysis of the five cases, including ours, we found that all the patients exhibited the common characteristic of slow bile drainage. Maroy’s case [6] presented an intrapancreatic bile duct with a greater stone above the pancreas. Ergul’s patient [5] had a blunt end of the CBD at the level of the ampulla of Vater. Mosenkis and Aftab presented mild dilation or even normal CBD [3, 4]. In our case, the end of the CBD was relatively narrow during the ERCP procedure. Due to stenosis at the distal end of the bile duct, bile and blood may empty slowly. Sandblom demonstrated that blood and bile do not mix with each other in the context of minor and slow bleeding. Solid clots subsequently form, which can lead obstructions [8]. Therefore, stenosis of the outflow tract may be conducive to the solidification of blood clots.

In contrast to bile duct obstructions caused by stones, obstructions caused by blood clots could be entirely attributed to their long oval shape and soft, dense, and elastic characteristics. Instead of completely removing the blood clot, Maroy inserted a plastic bile duct stent across the clot. Thus, inadequate drainage might be responsible for the patient’s delayed recovery.

Additionally, the time interval for the further ERCP will determine the severity of the complications. The sooner the treatment is performed, the quicker the patient will recover. Otherwise, complications might be more severe or even fatal. Ergül B’s post-ERCP cholangitis was noted the next day. The patient recovered well and was discharged 3 days after an emergency ERCP. In contrast, Aftab Ala’s ERCP was repeated 9 days after the initial procedure, and the patient recovered after 3 weeks.

In conclusion,delayed bleeding after ERCP cannot be avoided. Careful and gentle handling is necessary during the ERCP procedure. If the patient exhibits poor bile drainage due to anatomical factors, the use of endoscopic biliary stenting or ENBD to ensure continued biliary drainage is necessary. The diagnosis of blood clot is based on clinical and laboratory data, particularly imaging. Repeated ERCP is often necessary and effective.

## Abbreviations

ALT: Alanine aminotransferase
AST: Aspartate aminotransferase
CBD: Common bile duct
CT: Abdominal computerized tomography
ENBD: Endoscopic nasobiliary drainage
ERCP: Endoscopic retrograde cholangiopancreatography
EST: Endoscopic sphincterotomy
γ-GT: γ-glutamyl transpeptidase
